# Circadian Rhythms in Fractal Features of EEG Signals

**DOI:** 10.3389/fphys.2018.01567

**Published:** 2018-11-12

**Authors:** Pierpaolo Croce, Angelica Quercia, Sergio Costa, Filippo Zappasodi

**Affiliations:** ^1^Department of Neuroscience, Imaging and Clinical Sciences, G. d'Annunzio University, Chieti, Italy; ^2^Institute for Advanced Biomedical Imaging, G. d'Annunzio University, Chieti, Italy

**Keywords:** detrended fluctuation analyses, higuchi fractal dimension, circadian rhythm, amsterdam resting-state questionnaire (ARSQ), electroencephalography

## Abstract

Time-of-day modulations affect both performance on a wide range of cognitive tasks and electrical activity of the brain, as recorded by electroencephalography (EEG). The aim of this work was to identify fluctuations of fractal properties of EEG time series due to circadian rhythms. In twenty-one healthy volunteers (all males, age between 20 and 30 years, chronotype: neutral type) high density EEG recordings at rest in open and closed eyes conditions were acquired in 4 times of the day (8.00 a.m., 11.30 a.m., 2.30 p.m., 7.00 p.m.). A vigilance task (Psychomotor Vigilance Test, PVT) was also performed. Detrended fluctuation Analysis (DFA) of envelope of alpha, beta and theta rhythms was performed, as well as Highuchi fractal dimension (HFD) of the whole band EEG. Our results evidenced circadian fluctuations of fractal features of EEG at rest in both eyes closed and eyes open conditions. Lower values of DFA exponent were found in the time T1 in closed eyes condition, likely effect of the sleep inertia. An alpha DFA exponent reduction was found also in central sensory-motor areas at time T3, the day time in which the sleepiness can be present. In eyes open condition, HFD lowered during the day. In eyes closed condition, an HFD increase was observed in central and frontal regions at time T2, the time in which alertness reaches its maximum and homeostatic sleep pressure is low. Complexity and the persistence of temporal correlations of brain rhythms change during daytime, parallel to changes in alertness and performance.

## Introduction

According to the traditional model of control, physiological systems self-regulate their activity to preserve steadiness by reducing fluctuations around a homeostatic equilibrium point. Differently from this view, a wide bulk of evidence has recently been provided that several physiological time signals exhibit intrinsic fractal fluctuations (Goldberger et al., 2002; Stam, 2005). Indeed, heartbeat, respiration, gait rhythm, dynamics of neurotransmitter release, electromyography, brain activity reveal similar temporal patterns over multiple time scales (Hausdorff et al., 2001; Meyer and Stiedl, 2003; Fadel et al., 2004; Leao et al., 2005; Stam, 2005; Swie et al., 2005; He et al., 2007, 2010; Milstein et al., 2009; Scafetta et al., 2009; Zappasodi et al., 2015). An object exhibits fractal properties if similar details can be observed on different scales (Mandelbrot, 1983; Voss, 1988; Feder, 2013). These properties come up from nonlinear feedback interactions between mechanisms operating on multiple scales, sign of high integrity and adaptability of the whole system (Di Ieva et al., 2014). Also, a time process *X*(*t*) can display fractal properties if it possesses a scale-invariant structure over time and statistical similarity emerges at different time scales of its dynamics. For this process a self-affinity behavior can be retrieved (Barabasi and Albert, 1999), as *X*(*ct*) = *c*^*H*^ *X*(*t*). The estimation of the scaling exponent H, the Hurst exponent (Feder, 2013), has been found to be particularly attractive for describing the brain dynamics. Indeed, the amplitude modulation of oscillations of neuronal pools dynamics, revealed by electrophysiological techniques as electroencephalography (EEG) or magnetoencephalography (MEG), reveal long-term spatiotemporal structure in a temporal range from few seconds to tenth of minutes in resting state conditions at both eyes closed and eyes open (Linkenkaer-Hansen et al., 2001). The de-trended fluctuation analysis (DFA) is a widely-used method for the detection of long-range correlations in time series. Indeed, amplitude fluctuations of alpha, beta, and theta oscillations obey a power-law scaling behavior.

The fractal behavior of a time series has been linked to its “complexity,” that can be seen as the amount of information required to describe the time series (Mandelbrot, 1985). The concept of “complexity” refers to a highly structured temporal structure observed in the brain signal in an intermediate situation between pure randomness, like in white noise, and the absence of variability (constancy or pure periodicity), both conditions evidenced as non-physiological states (Di Ieva et al., 2014; Zappasodi et al., 2014; Smits et al., 2016). Since the existence of long term correlation is no guarantee of complexity, the complexity of a time series can be directly evaluated by its fractal dimension (Mandelbrot, 1985). Indeed, this measure quantifies the complexity and the self-similarity of a time series. The algorithm proposed by Higuchi (Higuchi fractal dimension, HFD, Higuchi, 1988) has been successfully directly applied to EEG signals to evidence modulation of complexity in different physiological conditions, as well as alterations in pathologies (for a review see Kesić and Spasić, 2016).

Fractal dimension and Hurst exponent quantify different properties: while the first is a local property, measuring the “roughness” of a signal (i.e., a “mild” or “wild” randomness), the latter quantifies a global characteristic, i.e., the long-memory dependence (long-range correlation) of the time series. For self-affine processes, where fractal properties can be retrieved, the local properties are reflected in the global one. Therefore, fractal dimension and Hurst exponent are linked.

Although the fractal properties have been recently described in brain time series both in health and disease, the neurophysiological mechanisms of fractal regulation are unknown. Recently the circadian pacemaker (suprachiasmatic nucleus) has been described to play a crucial role in generating fractal patterns in behavioral activity and heart rate at long time scales, and modulates their fluctuations at short time scales (Pittman-Polletta et al., 2013). Indeed, in humans, temporal fluctuations in physiological parameters and behavioral performance, on a wide range of cognitive functions, vary over the 24-h light-dark cycle. This cycle is driven by two interacting processes: the homeostatic sleep pressure (process S), which increases with time spent awake, and the circadian pacemaker (process C), a nearly 24-h endogenous process that drives at specific times of the day wakefulness and sleep (Borbély, 1982; Cajochen and Dijk, 2003; Rogers et al., 2003; Dijk and von Schantz, 2005; Cajochen et al., 2010). The circadian and homeostatic processes interact to provide stable levels of vigilance/alertness and cognitive performance during daytime (16-h) of normal wakefulness, when the circadian timing system fights the wake-dependent (or homeostatic) arousal decline. Indeed, alertness reaches its maxima during the early morning, when homeostatic sleep pressure is low, whereas decreases at its lowest level during the evening hours, when homeostatic sleep pressure is high (Van Dongen et al., 2003), even if exist a mid-afternoon window of sleep propensity (from ~14:00 to ~16:00) and an alertness window in the early evening hours from ~19:00 to ~22:00 (Lavie, 1989; Johnson, 1990). However, individual chronotype, namely “diurnal preference” in the timing of daily activities (Horne and Ostberg, 1976) under the control of the circadian clock (Roenneberg et al., 2007), influences peaks and troughs in alertness and performance. Hence, some people are more alert and perform better in the morning, whereas others in the evening, an effect referred to as the “synchrony effect” (May and Hasher, 1998) depending on individual chronotype.

In awake adults, data collected by sleep deprivation protocol and forced desynchrony protocol (i.e., sleep–wake cycle induced to uncouple from endogenous circadian rhythm) showed that both factors (process C and process S) contribute to a frequency-specific variation of EEG activity (Finelli et al., 2000; Cajochen and Dijk, 2003; Marzano et al., 2010). Moreover, resting waking EEG recordings are considered as an objective measure of alertness levels (Strijkstra et al., 2003). Specifically, an increase of EEG power density in the theta (4–8 Hz) and alpha (8–12 Hz) frequency range across prolonged periods of wakefulness has been associated with a decline of alertness and sleepiness (Drapeau and Carrier, 2004). In a recent EEG study, Meisel et al. (2017) reported a decline in long term correlation in alpha band as sleep deprivation progresses. Moreover, HFD has been applied to detect behavioral microsleep (Peiris et al., 2006) and changes from awake to drowsy states (Bojić et al., 2010; Pavithra et al., 2014).

The aim of this work was to identify fluctuations of fractal properties of EEG time series due to circadian rhythms. To this aim, high density EEG was collected in 4 different times of the day in both closed and open eyes conditions. Given the exploratory aspect of this study, we did not aim to differentiate the sleep pressure from endogenous factors, as usually done by using sleep deprivation or forced de-synchrony protocols but investigate if modulation of fractal properties over different day times can be retrieved in EEG at rest in physiological conditions.

## Methods

### Subjects

Twenty-one healthy volunteers (all males, age 23.6 ± 1.7) participated to the study. To avoid any kind of sleep debt and alterations of the sleep-wake cycle all selected participants reported no history of sleep, medical or psychiatric disorders and a good sleep quality (sleep schedule of 7-8 h/night), as assessed by self-rating questionnaires (Vignatelli et al., 2003; Violani et al., 2004). Moreover, in all participants, chronotype has been investigated by the Morningness Eveningness Questionnaire (MEQ, Horne and Ostberg, 1976), that assesses chronotype based on diurnal preferences (e.g., preferred time of day to perform physical and mental work; Horne and Ostberg, 1976). With this questionnaire, chronotype is categorized as a score (range:16–86), with high numbers corresponding to morning types (59 and above), low numbers corresponding to evening types (41 and below), and numbers between 42 and 58 corresponding to intermediate types. All selected participants had an intermediate chronotype (mean and standard deviation 53 ± 4). Exclusion criteria included shift workers, athletes and participants that had traveled crossing time zones in the 3 months before the study. The protocol was approved by the local Ethical Committee. All subjects gave written informed consent in accordance with the Declaration of Helsinki.

### Experimental procedure

For one week before the experiment, participants were asked to maintain a regular sleep-wake schedule. The night before the experiment, participants were asked to go to bed at their usual bedtime and to wake up at ~ 7:00 a.m. The quality of the sleep was checked by a wrist-worn actigraph (wActiSleep+, ActiGraph, Pensacola, FL, ActiGraph). A Sleep Efficiency >85% was required, to avoid any kind of sleep debt. These data were analyzed with Actilife (v.6.7.1, Actigraph^1^, Pensacola, FL), using a sleep/wake detection validated algorithm (Cole et al., 1992; Sadeh et al., 1994). Bed and rise times from the sleep diaries helped to frame the time in bed during which actigraphy data were analyzed.

The day of the experiment high density EEG recordings were acquired in 4 times of the day (T1: 8.00 a.m., T2: 11.30 a.m., T3: 2.30 p.m., T4: 7.00 p.m.) in 2 conditions: 10 min of eyes open and 10 min of eyes closed. The 2 conditions were randomized across subjects and times. The four times were chosen on the basis of well-known peaks of levels of vigilance/alertness and cognitive performance during daytime. Indeed, T0 correspond to the time were sleep inertia may be present (Jewett et al., 1999), in T2 alertness reaches its maxima and homeostatic sleep pressure is low, T3 is a mid-afternoon window of sleep propensity and in T4 homeostatic sleep pressure is high, but alertness is high (Lavie, 1989; Jewett et al., 1999; Van Dongen et al., 2003).

During the recordings, subjects were sitting on a comfortable armchair in a low light room and, in the eyes open condition, fixed a cross on a screen. Soon after both closed and open eyes recordings, participants were asked to complete the Amsterdam Resting-State Questionnaire (ARSQ). The questionnaire was presented on a screen and consisted of 55 statements about the feelings and thoughts experienced during the 10 min rest. For each statement, a 5-points rate from completely disagree to completely agree was used. Questions were grouped into 10 factors: Discontinuity of Mind, Theory of Mind, Self, Planning, Sleepiness, Comfort, Somatic Awareness, Health Concern, Visual Thought, and Verbal Thought (Diaz et al., 2013). Finally, a 10 min vigilance task (Psychomotor Vigilance Test, PVT; (Dinges and Powell, 1985) was done. Subjects were asked to fix a monitor with a red rectangular box and press a button when a counter appeared to the screen. The response stopped the counter and was required to be delivered as soon as possible. The period between the end of the counter and the begin of the following stimulus was randomly distributed between 2 and 10 s. To quantify the performance, the following parameters were extracted for each time (Basner and Dinges, 2011): number of lapses (i.e., number of responses >500 ms), number of false starts (i.e., response shorter than 100 ms), response speed (i.e., mean of the inverse of reaction times).

The EEG activity was recorded by a 128-channel system (Electrical Geodesic). The impedances were kept below 100 kΩ. EEG data were sampled at 250 Hz and collected for off line processing.

### Data analysis

Data were visually inspected to exclude saturated epochs of EEG signals from further analysis. A semi-automatic procedure, based on Independent Component Analysis (Barbati et al., 2004), was applied to identify and remove ocular, cardiac, and muscular artifacts. Signals were down-sampled to 125 Hz and re-referenced to the common average. Noisy channels were excluded and replaced by spline interpolation.

### Band power

The Power Spectral Density (PSD) was estimated for each EEG channel by means of the Welch procedure (Hamming windowing of 8 s, resulting in a frequency resolution of 0.125 Hz, 50% overlap). For each EEG channel and both conditions, band powers were obtained by the sum of the power spectrum in each frequency band normalized by the number of frequency bins. The considered frequency bands were: alpha (from 8 to 13 Hz), beta (from 15 to 25 Hz), and theta (from 4 to 7.5 Hz).

### Detrended fluctuation analysis

**Figure 1:**
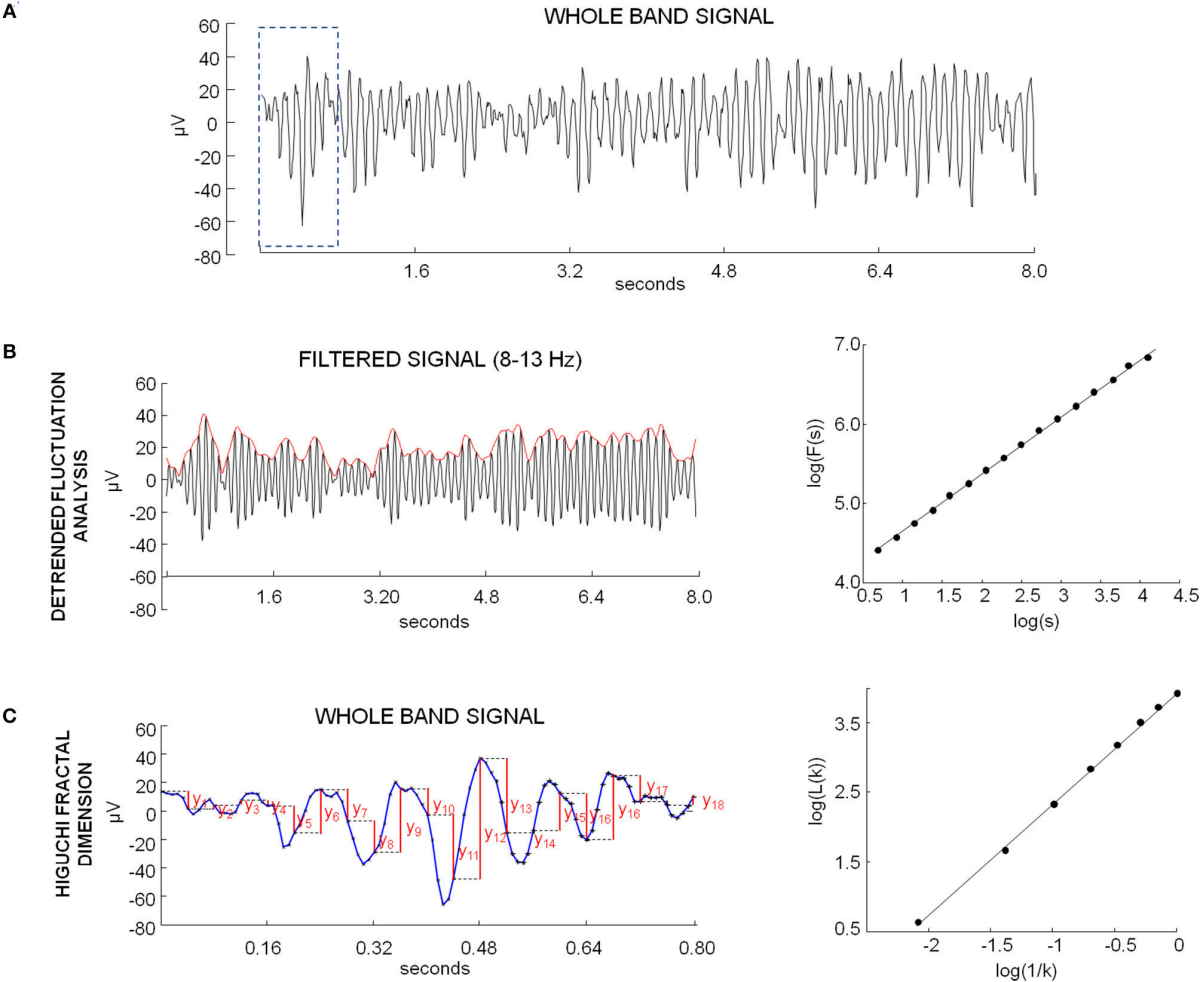
**(A)** Example of whole band EEG signal of one occipital channel in eyes closed condition. **(B)** Example of DFA calculation. Left: signal shown in **(A)** filtered in alpha band (black) and envelope obtained by modulus of analytic signal (red). Right: plot on logarithmic axes of the mean fluctuation per windows size, obtained from cumulative sum of alpha envelope, against the window size (expressed in seconds). The DFA exponent is the slope of the best-fit line. **(C)** Example of HFD calculation. Left: example sequence determination on the portion of EEG signal in the dotted box in A for the length calculation of $x_{k}^{m}$, with k = 5. The values are calculated as follows: |*x*(*m*+*i k*)−*x*(*m*+(*i*−1)*k*)|, for i = 1,2,3,…18. The length of the curve *L*(*k*) is evaluated as average over m of these values. Right: plot of *log*(*L*(*k*)) against *log*(1/*k*), for k = 1,. kmax (kmax = 8). The curve is said to have fractal dimension β if *L*(*k*)~*k*^−β^.

The DFA was applied to analyze the scale-free decay of temporal correlations in the amplitude envelope of brain rhythms. Peng et al. (1994) introduced this method to quantify long-range temporal correlation with less strict assumptions about the signal stationarity. The method quantifies the detrended fluctuations F(*n*) of the envelope at different time scales n. Firstly, each EEG signal was band-pass filtered in theta (4–7.5 Hz), alpha (8–13 Hz), or beta (15–25 Hz) band (Figures 1A,B). A Finite Impulse Response filter set to 2 cycles of the lowest frequency was used (filter order: 62 for theta, 31 for alpha, 16 for beta band). The envelope of the band-passed signals was computed by the modulus of its analytic signal, obtained by Hilbert transform. The cumulative sum *y* of the envelope *x* was then calculated:

$$\begin{array}{l} y\left(k\right)=\;\overset{k}{\underset{i=1}{\sum }}|x\left(i\right)-<x>| \end{array}$$

Where, <*x*> denotes the mean of the envelope *x*. By applying scaling analysis to *y*(*k*) no a priori assumptions about the signal stationarity is required (Hardstone et al., 2012).

The cumulative sum was then partitioned into *N*_*s*_ windows of length *s (j* = 1, 2, …*N*_*s*_). For each window, the local trend was calculated by a least-square line fitting procedure (Kantelhardt et al., 2002). If x_j, s_(i) is the ordinate of the fitting line of the j-th segment of length s at time bin *i* (*i* = 1,2,…*s*), the fluctuation of the j-th segment of length *s*, i.e., the root-mean-square deviation from the trend, was calculated as:

$$\begin{array}{l} RMS_{j}^{s}=\frac{1}{2}\overset{s}{\underset{i=1}{\sum }}{\left\{x\left[\left(j-1\right)s+i\right]-x_{j,s}\left(i\right)\right\}}^{2} \end{array}$$

To obtain the fluctuation function, for each scale *s* the average of the root mean square deviation from the trend was obtained (Kantelhardt et al., 2002):

$$\begin{array}{l} F\left(s\right)=\;\sqrt{\frac{1}{N_{s}}\overset{S}{\underset{j=1}{\sum }}RMS_{j}^{s}} \end{array}$$

The scaling behavior of the fluctuation function can be obtained by the log-log plot of *F*(*s*) over *s* (Figure 1B). If a long-range power-law correlation exists, the following relationship holds:

$$\begin{array}{l} F\left(s\right)\sim s^{H} \end{array}$$

and the plot is a line, with slope equal to H, the DFA exponent or Hurst exponent (Feder, 2013).

The inclusion of very short windows in the fitting range of the fluctuation function introduces correlation between neighboring samples of the signal. For this reason, we applied the procedure presented in Hardstone et al. (2012) to estimate the effect of narrow-band filtering in DFA values for theta, alpha and beta bands. Briefly, for each band 1000 realizations of white noise were generated and band-pass filtered. On each signal, the amplitude envelope was extracted, DFA performed and the lowest fitting time window estimated from the log-log plot of scale against fluctuation function. The investigated scale ranges from 0.1 s to 100 s. Since for a white-noise signal a DFA exponent of 0.5 is expected, the lowest fitting time window was chosen as the value of the scale after that the trend line of the fluctuation function has a slope of 0.5. Based on obtained results, we found a lowest fitting time window of 2 s for alpha band, of about 1.4 s for beta band and of 4 s for theta band. Therefore, we estimated DFA exponent with a scale in a range of 2 s to 1 min for alpha and beta band and of 4 s to 1 min for theta band.

### Higuchi fractal dimension

For both conditions and in each time, HFD values of each EEG channel were computed for the whole band signal (i.e., signal filtered between 1 and 40 Hz). As a global measure of HFD, all HFD values obtained for the single channels were averaged.

Fractal dimension is considered as a measure of complexity of a curve. For time series representing this curve, HFD ranges from 1 for deterministic constant functions to 2 for white noise. The algorithm proposed by Higuchi was used (Higuchi, 1988; Accardo et al., 1997). Briefly, the algorithm directly estimates the mean length of the curve *L*(*k*) through a measure unit of a segment of *k* samples. From any given time series of *N* samples: *x*(1), *x*(2), …*x*(*N*), *k* new time series with initial time sample *m* and time step *k* are derived as:

$$\begin{array}{l} x_{k}^{m}:\;x\left(m\right),x\left(m+k\right),x\left(m+2k\right),\;\ldots x\left(m+int\left(\frac{N-m}{k}\right)k\right) \end{array}$$

The length of each curve $x_{k}^{m}$ is calculated as follows (Figure 1C):

$$\begin{array}{l} L_{m}\left(k\right)=\frac{1}{k}\left[\frac{N-1}{int\left(\frac{N-m}{k}\right)k}\left(\overset{int\left(\frac{N-m}{k}\right)}{\underset{i=1}{\sum }}|x\left(m+i\;k\right)-x\left(m+\left(i-1\right)k\right)|\right)\right] \end{array}$$

For each k, the length of the curve *L*(*k*) is evaluated as:

$$\begin{array}{l} L\left(k\right)=\frac{1}{k}\overset{k}{\underset{m=1}{\sum }}L_{m}\left(k\right) \end{array}$$

The calculation of the curve length *L*(*k*) is repeated for *k* from *1* to *k*_*max*_. The curve is said to have fractal dimension β if:

$$\begin{array}{l} L\left(k\right)\sim k^{-\beta } \end{array}$$

In this case the plot of *log*(*L*(*k*)) against *log*(*k*) should fall on a straight line with slope equal to –β, so HFD can be obtained by a least-squares linear best-fitting procedure (Figure 1C).

Since HFD is highly dependent on the value of K_max_, this parameter has a crucial role in HFD estimation. In our knowledge, the studies addressing this issue tested different values directly on their data (for a review see the Appendix A in Kesić and Spasić, 2016). For this reason, according to previous studies (Zappasodi et al., 2014, 2015), a value of k_max_ = 8 was applied to the whole-band EEG with a sampling frequency of 125 Hz.

### Statistical analysis

The aim of the statistical analysis was to test if differences across times were present in the non-linear fractality measures depending on the condition (eyes open or closed). Firstly, the topographies of both Hurst exponent and HFD were obtained separately for each band in all the 4 times and the 2 conditions. For each subject, each time and each condition, in the topographical maps the channels of maximum amplitude have been chosen and the channels around the maxima with values exceeding the 90% of maximum have been individuated. Clusters of electrodes have been obtained by conjunctions of these groups of electrodes. The mean Hurst exponent and HFD values over these channels were considered for further analysis. Repeated measure Analyses of Variance (ANOVAs) were separately performed for Hurst exponent and HFD. A 4 X 2 X N design was applied, with Time (T1, T2, T3, T4), Conditions (eyes open, eyes closed) and Region (N maxima individuated on the topographies) as within-subject factors. Greenhouse-Geisser correction has been applied if the sphericity assumption was not valid. *Post-hoc* paired samples *t*-tests were carried out to assess significant differences among times. *Post-hoc* comparisons were FDR corrected.

DFA exponent values could depend on band power. Indeed, estimates of DFA exponent can be biased toward lower values when amplitude of the rhythm reduces, and signal-to-noise ratio increases and vice-versa toward higher values when amplitude increases. Therefore, Spearman's correlations between DFA exponent and corresponding band powers were calculated to evidence positive correlations. Moreover, the same ANOVA design of DFA and HFD was applied on band powers, by considering regions with the same channels used for the fractal measures.

Finally, to verify if band power of rest EEG or non-linear measures (DFA exponents or HFD) predict ARSQ factors, multiple regression analysis was separately performed on each ARSQ factor, considered as dependent variables of the model. Values of each time and both conditions were considered together (4 times X 2 conditions X 20 subjects, resulting in 160 variables). Independent variables were all the band powers, DFA exponents and HFD values in the considered regions. Times and conditions were also included in the model as categorical variables.

## Results

**Figure 2:**
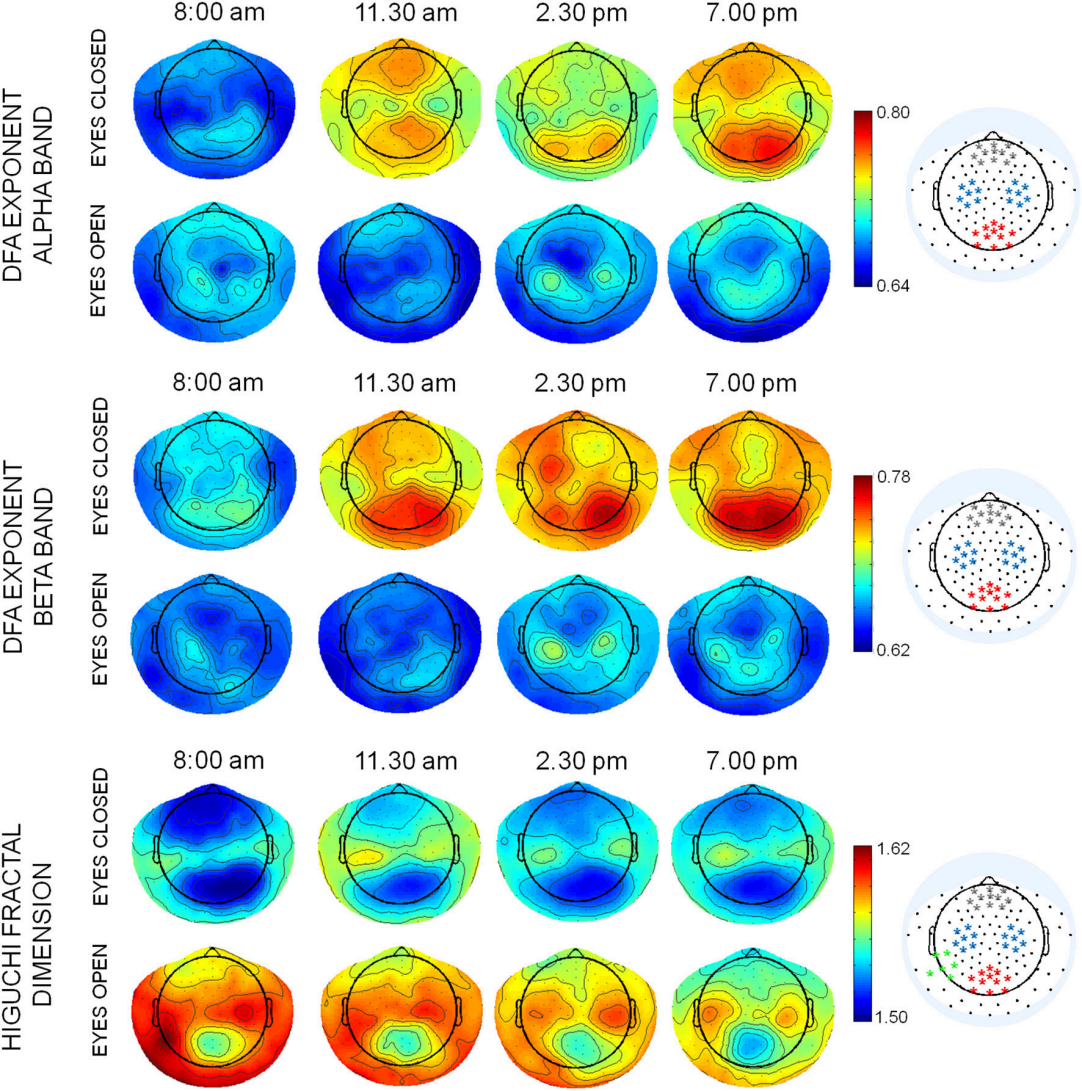
Mean topographies of DFA exponents in alpha and beta bands and of HFD values in eyes closed and open conditions in the 4 times T1: 8:00 am; T2: 11:30 am, T3: 2:30 pm; T4: 7:00 pm. The selection of channels used for averaging the DFA and HFD values are displayed on the right (red: posterior parieto-occipital region; blue: central sensorymotor regions; gray: frontal region; green: left temporo-parietal region).

The mean topographies of DFA exponent of alpha and beta bands and of HFD were similar across the four times, with the maximal values in specific regions, depending on condition. In alpha band, maximal values were located in occipito-parietal and frontal regions, in particular in eyes closed condition, while in eyes open condition maxima of DFA exponents were also observed in bilateral sensorimotor regions (C3 and C4 of the 10–20 international system, Figure 2). In beta band, the posterior maximum of DFA exponents in eyes closed and central areas in eyes open condition were found approximately on the same electrodes of alpha band. The mean topographies of HFD values showed maxima in central sensorimotor regions and minima in parieto-occipital and frontal regions. A maximum of HFD was observed in the temporo-parietal electrodes of left hemisphere (T5 and TP9) in eyes open condition. Channels around the maxima of the posterior, frontal, and bilateral central areas were chosen to average DFA exponents in alpha and beta bands (Figure 2) for the ANOVA design. The same channels were chosen for HFD values, with in additions channels around the left temporo-parietal maximum. In theta band, no specific topographies of DFA exponents over the 4 times were observed. Therefore, to assess whether DFA exponents changed over times, a global DFA theta value was calculated for each subject and both condition as the mean over all EEG channels.

### Detrended fluctuation analysis

Fifth, ninety-fifth percentile of Hurst exponent values ranged from 0.57 to 0.86 in alpha band (mean ± standard deviation across subjects and times: 0.73 ± 0.09 and 0.69 ± 0.08, respectively for closed and open eyes), from 0.57 to 0.85 in beta band (0.72 ± 0.09 and 0.67 ± 0.07, respectively for closed and open eyes), and from 0.53 to 0.74 in theta band (0.62 ± 0.06 and 0.60 ± 0.07, respectively for closed and open eyes). All mean DFA values were significantly different from 0.5, the DFA exponent value of uncorrelated white noise (one-sample *t*-test *p* < 0.0001 for each band, region, and condition).

**Figure 3:**
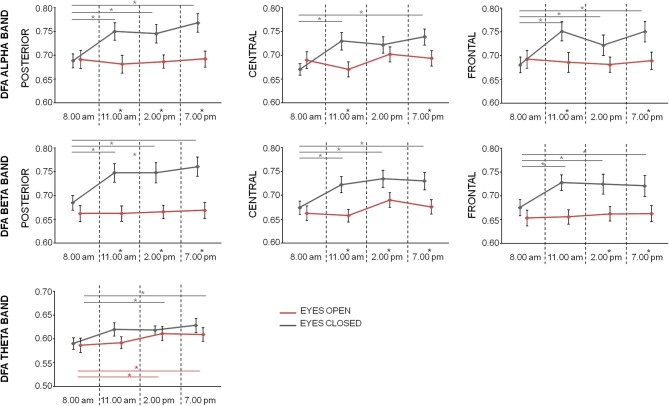
Mean (bars indicate standard error of mean) of DFA exponent values in the different regions for alpha, beta, and theta bands at the 4 times in closed eyes (gray) and open eyes (red) conditions. Significances of the paired *t*-test between times are shown (gray for closed eyes and red for open eyes; ^*^p < 0.05, FDR corrected). The difference between eyes closed and eyes open conditions at one time is evidenced by a star as apex of corresponding time.

In alpha band, repeated measures ANOVA with *Time* (four levels: T1, T2, T3, and T4), *Condition* (two levels: Eyes Closed, Eyes Open), and *Region* (three levels: posterior, central, frontal) as within-subject factors showed significant main effects of *Time* [*F*_(3, 60)_ = 3.492; *p* = 0.021] and *Condition* [*F*_(1, 20)_ = 5.808; *p* = 0.026], as well as significant Condition^*^Time [*F*_(3, 60)_ = 4.183; *p* = 0.009], and Condition^*^Region [*F*_(1.5, 29.8)_ = 4.218; *p* = 0.034] interaction effects, but not significant main effect of *Region* (*p* = 0.301) and interactions Region^*^Time (*p* = 0.101) and Condition^*^Region^*^Time (*p* = 0.718). Looking at the alpha DFA exponent over time (Figure 3), we noticed that at T1 no differences were observed between conditions in all regions. The marked differences were present only at time T2, T3, and T4 for occipital regions and T2 and T4 for frontal and central regions, as assessed by paired *t*-test between eyes closed and eyes open conditions (*p* < 0.05, FDR corrected, Figure 3). Moreover, while in open eyes condition no difference among DFA exponent in the different times was found, in closed eyes condition DFA exponent values at time T1 was lower than values at other times in occipital and frontal regions (paired sample *t*-test between T1 and the other times consistently *p* < 0.05, FDR corrected). In central regions, only differences between T1 and T2 and between T1 and T4 were observed.

In beta band, repeated measures ANOVA with *Time, Condition*, and *Region* as within-subject factors showed significant main effects of *Time* [*F*_(3, 60)_ = 4.128; *p* = 0.010], *Condition* [*F*_(1, 20)_ = 14.319; *p* = 0.001], *Region* [*F*_(2, 40)_ = 9.433; *p* < 0.001] as well as significant Condition^*^Region [*F*_(1.3, 27.0)_ = 4.222; *p* = 0.039] and Region^*^Time [*F*_(6, 120)_ = 2.234; *p* = 0.044] interaction effects. No significant interactions Condition^*^Time was found [*F*_(3, 60)_ = 2.526; *p* = 0.066] and Condition^*^Region^*^Time (*p* = 0.374). Also, in this band, DFA exponent values in the eyes open conditions did not differ over time. On the contrary, in closed eyes condition, T1 values were lower than values at other times in all regions (*p* < 0.05 consistently, FDR corrected). Differences between the conditions were observed in all regions at T2, T3, and T4 (*p* < 0.05, FDR corrected).

Finally, Repeated measures ANOVA on theta DFA values with *Time* and *Condition* showed only a significance of the main effect *Time* [*F*_(3, 60)_ = 4.669; *p* = 0.005]. The lack of Condition effect (*p* = 0.155), or interaction Time^*^Condition (*p* = 0.428), indicated that in the 4 times the theta DFA exponents were not different between open closed and eyes conditions. *Post-hoc t*-test indicated a difference between T1 vs. T3 and T1 vs. T4 (*p* < 0.05, Figure 3).

### Higuchi fractal dimension

Fifth, ninety-fifth percentile of HFD values ranged from 1.44 to 1.69 (mean ± standard deviation across subjects and times: 1.54 ± 0.08 and 1.59 ± 0.08, respectively for closed and open eyes).

**Figure 4:**
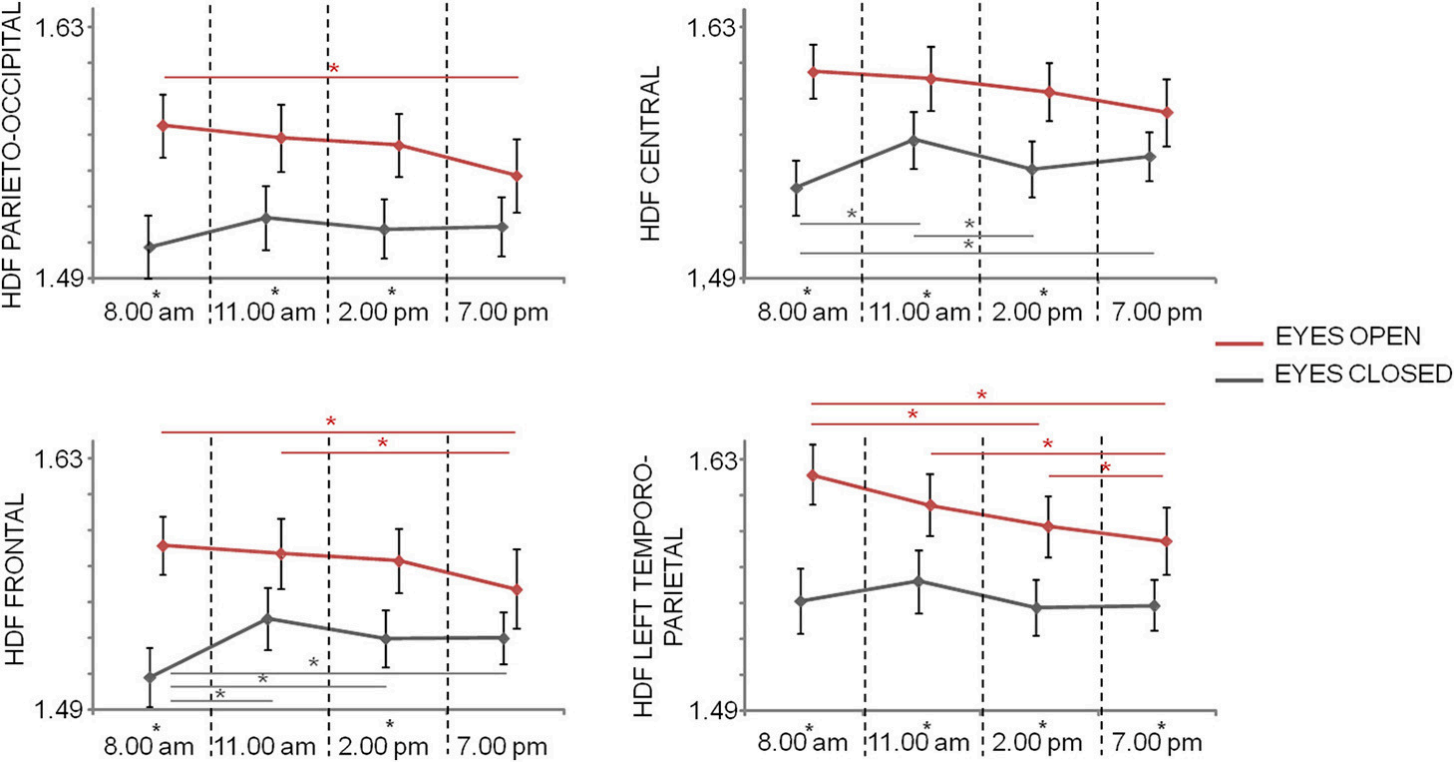
Mean and standard error of HFD values in the different regions at the 4 times in closed eyes (gray) and open eyes (red) conditions. Significances of the paired *t*-test between times are shown (gray for closed eyes and red for open eyes; ^*^p < 0.05, FDR corrected). The difference between eyes closed and eyes open conditions at one time is evidenced by a star as apex of corresponding time.

Repeated measures ANOVA with *Time, Condition*, and *Region* (four levels: posterior, central, frontal, left temporo-parietal) as within-subject factors showed significant main effects of *Condition* [*F*_(1, 20)_ = 21.193; *p* < 0.001] and *Region* [*F*_(3, 60)_ = 19.608; *p* < 0.001], as well as significant Condition^*^Time [*F*_(3, 60)_ = 7.280; *p* < 0.001], Region^*^Time [*F*_(5.0, 100.6)_ = 2.933; *p* = 0.016] interaction effects, but not significant main effect of *Time* [*F*_(3, 60)_ = 2.319; *p* = 0.084], and interactions Region^*^Condition (*p* = 0.405) and Condition^*^Region^*^Time (*p* = 0.932). In parieto-occipital and central regions differences between open and closed eyes conditions were observed in T1, T2, and T3 times (*p* < 0.05), with eyes open HFD values higher than eyes closed HFD values. This difference was also present in all times in left temporo-parietal regions and at times T1 and T3 in frontal regions. Moreover, while in central regions eyes open HFD values did not significantly changed over times, in the other condition a reduction was observed over time (Figure 4). In eyes closed condition, an increase at T2 and T4 with respect to T1 and T3 were observed in central regions and the T1 values were lower than the values at the other times in frontal regions (Figure 4).

### Band power

Repeated measures ANOVA with *Tim*e, Condition, and Region on alpha band power values revealed significant main effects of *Condition* [*F*_(1, 20)_ = 28.764; *p* < 0.001], *Region* [*F*_(3, 60)_ = 64.231; *p* < 0.001], and *Time* [*F*_(3, 60)_ = 4.874; *p* = 0.004]. The first 2 main effects confirmed that alpha power in closed eyes condition were higher than in eyes open condition and that alpha power was higher in parieto-occipital regions. The lack of interactions Time^*^Condition (*p* = 0.574), Time^*^Regions (*p* = 0.311), Time^*^Condition^*^Regions (*p* = 0.549) and the presence of the main effect *Time*, confirmed that during the day the alpha power uniformly changed in all the considered groups of electrodes (mean and standard error over conditions and regions: 6.29 ± 0.10 at T1, 6.38 ± 0.09 at T2, 6.40 ± 0.09 at T3, 6.42 ± 0.10 at T4). In particular, *post hoc* tests showed that the difference was significant only between T1 and T4 (*p* = 0.036 Bonferroni corrected). The same results were found in beta band: the main significant effects *Condition* [*F*_(1, 20)_ = 8.062; *p* = 0.010], *Region* [*F*_(3, 60)_ = 49.261; *p* < 0.001], and *Time* [*F*_(3, 60)_ = 8.643; *p* < 0.001] and the lack of interactions (*p* > 0.1) confirmed a similar increase of power (5.50 ± 0.07 at T1, 5.60 ± 0.07 at T2, 5.62 ± 0.07 at T3, 5.63 ± 0.07 at T4). *Post hoc* tests showed differences of beta band at T1 vs. T2 (*p* = 0.009), at T1 vs. T3 (*p* = 0.029), at T1 vs. T4 (*p* = 0.007). Finally, repeated measures ANOVA with *Tim*e and *Condition* on theta band power showed only a significant main effect of *Time* [*F*_(3, 60)_ = 3.492; *p* = 7.834]. Also, for this band, T1 values were different by T2, T3, and T4 (*p* = 0.042, *p* = 0.018 and *p* = 0.011, respectively; mean and standard over conditions: 5.99 ± 0.08 at T1, 6.11 ± 0.08 at T2, 6.15 ± 0.08 at T3, 6.16 ± 0.07 at T4).

In closed eyes condition, no correlations between DFA exponent and band powers were observed for alpha and beta bands. A positive correlation was found for theta band. On the contrary, positive correlations were found between all the band powers and DFA exponent in all bands in eyes open condition (Table 1).

**Table 1:** Rho (*p*-values in italics, not corrected for multiple comparisons) of Spearman's correlations between DFA exponents and band powers in eyes closed and eyes open conditions.

	**Eyes Closed**	**Eyes Open**
Posterior alpha	0.111	0.462
	*0.374*	*<0.001*
Central alpha	0.115	0.422
	*0.310*	*<0.001*
Frontal alpha	0.079	0.478
	*0.486*	*<0.001*
Posterior beta	0.184	0.352
	*0.102*	*<0.001*
Central beta	0.188	0.421
	*0.094*	*<0.001*
Frontal beta	0.194	0.425
	*0.085*	*<0.001*
Theta	0.455	0.684
	*<0.001*	*<0.001*

### Psychomotor vigilance test

The numbers of lapses and false starts did not change over time and was lower or equal to 1 for each conditions and subject. Repeated measure ANOVA design on response speed indicated a significant *Time* effect. Indeed, T4 speed was higher than T1 (*p* = 0.012, Bonferroni corrected) and tended to be higher also than the T3 speed (*p* = 0.085). Mean (standard deviation) values of response speed were (in s^−1^): 3.574 ± 0.231 at T1; 3.639 ± 0.387 at T2; 3.568 ± 0.330 at T3; 3.678± 0.288 at T4.

### Amsterdam resting-state questionnaire

Repeated measure ANOVA with *Time* and *Condition* as within subject factors separately applied to ARSQ factors, revealed significant effects only for *Sleepiness* and *Somatic Awareness*. In particular, in Sleepiness a significant effect of the main factor *Time* [*F*_(3, 54)_ = 4.992; *p* = 0.004] and an interaction Condition^*^Time [*F*_(3, 54)_ = 3.101; *p* = 0.034] were found, but not the main effect of *Condition* (*p* = 0.877). *Post-hoc* tests revealed that, while no differences in times were found in open eyes condition (mean values ± standard deviation for T1, T2, T3, and T4: 1.67 ± 0.20; 2.13 ± 0.25; 2.22 ± 0.26; 1.88 ± 0.22), T1 scores were lower than the scores at the other times in eyes closed condition (T1: 1.27 ± 0.19; T2: 2.02 ± 0.26; T3: 2.26 ± 0.25; T4: 2.19 ± 0.25, paired-sample *t*-test T1 vs. T2, *p* = 0.009; T1 vs. T3, *p* = 0.006; T1 vs. T4, *p* = 0.010, Bonferroni corrected). For Somatic Awareness, only the significance of the main factor *Time* was found [*F*_(3, 54)_ = 3.661; *p* = 0.009] and neither *Condition* (*p* = 0.475) nor the interaction Condition^*^Time (*p* = 0.862) resulted significant. *Post-hoc* tests revealed a difference only between T1 and T4 times (*p* = 0.050, mean scores between the 2 conditions: 2.14 ± 0.14; 1.98 ± 0.16; 1.85 ± 0.15; 1.69 ± 0.18 at the 4 times).

### Relationship between spectral and fractal features with ARSQ factors

As shown in Table 2, different spectral and fractal features entered the regression model, predicting factors of ARSQ. Up to the 27% of the variance was explained (minimum 9% for *Sleepiness*, maximum 27% for *Somatic Awareness*). A positive sign of the estimated coefficients indicates a positive relationship between the spectral/fractal variables and the ARSQ factors, i.e., the higher the power band or fractal measure, the higher the score of ARSQ. A negative sign indicated the opposite. No variables entered the model for the *Comfort* factor.

**Table 2:** Regression models with ARSQ factors as dependent variables and spectral/fractal features as independent variables.

**Dependent variables**	**Independent variables**	**Estimated coefficients**	***R*-values**	**ANOVA**
Discontinuity of Mind	Left temp Beta Pow	−1.157		*F*_(3, 154)_ = 11.369; *p* < 0.001
	Theta Pow	0.776	0.425	
	Central DFA exp - alpha	1.428		
Theory of Mind	Left temp Beta Pow	−1.326	0.438	*F*_(2, 155)_ = 18.434; *p* < 0.001
	Theta Pow	0.603		
Self	Frontal HFD	2.970		
	Central Beta Pow	−1.121	0.475	*F*_(3, 154)_ = 14.973; *p* < 0.001
	Posterior Beta Pow	0.651		
Planning	Central Beta Pow	−0.796		
	Posterior Beta Pow	1.297	0.406	*F*_(3, 154)_ = 10.154; *p* < 0.001
	Left temp Beta Pow	−1.013		
Sleepiness	Central DFA exp – beta	3.005	0.308	*F*_(2, 155)_ = 8.108; *p* < 0.001
	Time	0.157		
Comfort	No variables entered			
Somatic Awareness	Frontal DFA exp - alpha	−0.755		
	Posterior Alpha Pow	−0.178	0.524	*F*_(5, 152)_ = 11.537; *p* < 0.001
	Posterior HFD	9.829		
	Frontal HFD	−6.298		
	Time	−0.103		
Health Concern	Central HFD	1.854		
	Frontal DFA exp—alpha	−1.463	0.350	*F*_(3, 154)_ = 7.182; *p* < 0.001
	Condition	−0.216		
Visual Thought	Left temp Beta Pow	−0.937		*F*_(3, 154)_ = 8.200; *p* < 0.001
	Theta Pow	2.101	0.372	
	Central Beta Pow	−1.345		
Verbal Thought	Left temp Beta Pow	−2.051		*F*_(3, 154)_ = 14.853; *p* < 0.001
	Central HFD	−3.007	0.473	
	Theta Pow	1.058		

*Times and conditions were included as categorical variables*.

## Discussion

Our results evidenced circadian modulations of fractal features of EEG at rest in both eyes closed and eyes open conditions. The investigated fractal characteristics were long term memory of amplitude modulation of alpha, beta, and theta rhythms in a time range from few seconds to 1 min, as assessed by Hurst exponent estimated by Detrended Fluctuation Analysis, and global complexity, as assessed by Higuchi Fractal Dimension.

According to previous results (Linkenkaer-Hansen et al., 2001; Nikulin and Brismar, 2004, 2005), in our study scaling exponent values (approximately between 0.6 and 0.8) indicated the presence of long range temporal correlation of neuronal oscillations in alpha, beta and theta bands. The presence of high long-term correlation indicates a more temporally structured amplitude modulation of the neuronal rhythms, building up through neural local interactions until they extend throughout the whole system (Linkenkaer-Hansen et al., 2001). It is not yet understood if the less temporally uncorrelated modulation of the rhythm is a sign of a reduced functionality of the brain areas. It has been suggested that the temporal correlations of amplitude modulation of oscillations on time scales of seconds to tens of seconds may be important for the temporal integrity of cognition, since a reduction of scaling exponent is related to several neurological impairments and diseases (Hausdorff et al., 2001; Parish et al., 2004; Linkenkaer-Hansen et al., 2005; Montez et al., 2009). Moreover, a more whitened state, characterized by a lower scaling exponent, has been found to lead more often to percept destabilization (Sangiuliano Intra et al., 2018).

Topography of DFA exponents confirmed a scalp distribution coherent with the physiological distribution of cortical rhythms, in posterior occipital-parietal, central sensory-motor, and frontal regions. This distribution indicates a topographical specialization of brain areas. Indeed, all cortical circuits accumulate information over time to continuously use past information to process the incoming one. As shown in an electrocardiographic study (Hasson et al., 2015), the timescale of accumulation, linked to the scaling exponent, changes hierarchically from short processing timescale, typical of sensory regions, to higher-order regions, which show typically long processing timescales.

No differences in eyes open conditions over day times were observed in our data. On the contrary, lower values of DFA in both alpha and beta bands were observed in eyes closed condition in the first time, between 8.00 and 9.00 a.m. In this line, the significantly lower values of DFA exponent we found in the time T1 may be the expression of a lowered arousal, causing a decrement in cognitive performances in the first hours of the morning, due to effects of the sleep inertia (Jewett et al., 1999; Ferrara and De Gennaro, 2000). This reduction in T1 was observed only in closed eyes condition, in which it is likely that arousal levels were lower. In line with this interpretation, we found that the PVT performance was lower in the time T1, suggesting a reduced vigilance in this time. A reduction in beta activity upon awakening has been previously found in EEG recording as sign of sleep inertia (Marzano et al., 2011). Interestingly, an alpha scaling exponent reduction was found also in central sensory-motor areas at time T3 in closed eyes condition. In our data, the maximum of mean values of sleepiness factor in the ARSQ, also if not reaching significance, was reached in the T3 time. These results may be interpreted as a reduced functionality in the day time in which the sleepiness can be present (Jewett et al., 1999).

In a recent EEG study, Meisel et al. (2017) found in a sustained wakefulness protocols, a decline of scaling exponent in alpha band as sleep deprivation progresses, apparently contrary to our finding of a DFA exponent increase during the day. However, the aims of this study were different from ours, as subjects were sleep deprived. We tried to keep the physiological conditions as ecological as possible: subjects were outside the laboratory between 2 consecutive measures, the light exposure was natural, and, under the supervision of an experimenter, they were in their habitual environment. The differences between the 2 studies may be caused also by individual variation in the circadian influence on fractal neural activity control (21 subjects in our study, 7 subjects in Meisel et al., 2017).

As previously pointed out (Kantelhardt et al., 2002; Hardstone et al., 2012), power amplitude could bias the values of DFA exponent, since low amplitude could be associated to low signal to noise ratio, and scaling exponent could be reduced toward values more similar to scaling exponent of white noise. On the contrary, high amplitude, resulting in high signal to noise ratio, could bias toward higher values of DFA exponents. For this reason, we investigated also the effect of time on power bands. We found both in eyes open and eyes closed conditions, an increase in all bands over time. However, the lack of correlation between DFA exponent values and alpha and beta band powers in eyes closed condition confirmed that our results on DFA exponents are not due to the increase of band power. On the contrary, in theta band, no difference between eyes closed and eyes open condition was found and a similar trend between scaling exponent and power was evidenced. Since a high correlation between the scaling exponent and the theta power was found, we cannot exclude that the results in theta band may be biased by the power changes over time.

Our results in band power are in accordance to previous studies. In protocols with 40 h sustained wakefulness, theta band exhibited a minimum ~1 h after the onset of melatonin secretion and alpha band activity showed a minimum close to the body temperature minimum (Aeschbach et al., 1999), therefore minima of daily theta and alpha activity were found in the first hours of the morning. In these studies, both circadian effects and endogenous processes interact. In contrast, in forced desynchronized paradigms, where subjects were kept several days in an environment free of time cues with an artificial dim light, the circadian rhythm of plasma melatonin desynchronized and the contribution of circadian phase (process C) can be separated by the elapsed time awake effects (process S). In this situation, effects on EEG band power in wakefulness of both processes have been described (Cajochen et al., 2002). Specifically, circadian oscillations of theta, alpha, and beta bands have been found, with increase during the daytime and decrease during the biological night hours. Minimum of beta and theta activity was in correspondence of the onset of melatonin secretion, located in fronto-central derivation, and the minimum of alpha activity in posterior and frontal regions was close to the peak of melatonin rhythm (Cajochen et al., 2002). Our findings in band power are in line with these results, since an increase during the day was observed, with minima in the first T1 time. Wake-dependent variations in desynchronized protocols are more pronounced in frontal regions, with an increase of beta band. A reduction of alpha activity with elapsed time awake was also observed (Cajochen et al., 2002; for a review see Cajochen and Dijk, 2003).

Our data showed changes during the daytime also on HFD in a spatial-dependent modality, depending on condition (eyes closed or open). Indeed, in eyes open condition, fractal dimension lowered during the day, in particular in occipital, frontal, and temporo-parietal regions. In eyes closed condition, an HFD increase was observed at time T2 in central and frontal regions. Decrease of complexity over time during the day in the open eyes condition may be interpreted as a circadian modulation of efficiency of neural activity parallel to changes in arousal and cognitive performance (Wright et al., 2012). In line with this interpretation, an increase of complexity in central and frontal areas was found at T2, the time in which alertness reaches its maximum and homeostatic sleep pressure is low.

The fluctuations over daytime of fractal features we observed, tend to implicate that the complexity of brain electrical activity cannot be entirely described by a single scaling exponent. This may suggest a multi-fractal nature of brain dynamics. Indeed, previous studies evidenced multi-fractal nature of the human sleep EEG activity (Ma et al., 2006; Weiss et al., 2009, 2011), and showed that multifractality might be an adequate approach for compact modeling of brain activities and a useful pattern classification technique to distinguish among different brain states during sleep (Weiss et al., 2011; Zorick and Mandelkern, 2013). Future studies with an extensive characterization and a detailed topographic analysis of EEG multi-fractal features in awake human EEG are needed to systematically address this point.

A direct causal influence of circadian rhythm to scaling properties cannot be supported by our data. Indeed, scaling would be the result of stochastically perturbed oscillatory entrapment across a broad range of times scales (Bak et al., 1987; Turcotte, 1999), and circadian rhythms could come out from a background of stochastically fluctuating biological processes at different temporal scales. From a theoretical perspective, this view would overturn the more intuitive notion that very regular biological oscillations regulate physiology, and regulate also scaling, in favor of the idea that scaling itself is the background model for the dynamics of physiological time series and thus also for their fluctuations at different time scales. In this context, we can hypothesize that homeostatic sleep pressure, together with other endogenous and exogenous physiological factors (Muto et al., 2016), contribute to brain dynamics, characterized by a fractal, or even better multi-fractal, behavior. As a result, daily fluctuations of scale exponents and complexity can be found in brain dynamics.

The question arises as to whether fractal dimension and Hurst exponent provide additional information to spectral features in describing the rest conditions. Previous studies reported evidence that variation in spectral and fractal feature of EEG can be linked to retroactive self-reports of subjective experiences at rest (Knyazev, 2013; Diaz et al., 2016). Even if with an exploratory purpose, we separately performed a regression analysis for each factor of ARSQ, considered as dependent variable, and with fractal dimension, scaling exponents and band powers as independent variables. We found that not only spectral features, but also fractal characteristics entered the model to explain up to the 20% of the variance. These relationships are suggestive of the ability of fractal features to summarize the neuronal activity in terms of temporal structuring or complexity in relation to cognition or behavior. In particular, reduction of left temporo-parietal or central beta power and increase of theta activity was linked to higher scores of several ARSQ factors, underling the role of beta desynchronization/theta synchronization of these rhythms in several cognitive domains (Engel and Fries, 2010). Positive signs of the estimated coefficients of the regression models were found for beta DFA exponents and HFD values. This finding indicates that increase in complexity in specific areas or a greater persistence of temporal correlations in alpha or beta bands predict higher score of specific ARQS factor. A negative coefficient was found only for frontal alpha DFA exponent in Somatic Awareness and Health Concern. Irrmischer et al. (2018) found an increase of Somatic Awareness during meditation and a decrease of alpha scaling exponent most pronounced above parietal, central, and frontal regions, but also a decrease in Health Concern was found. A direct link between ARSQ factor and spectral or fractal features is beyond the aim of this work. The interesting finding here is that our results underline the fact that spectral features cannot be considered alone in explaining highly non-linear phenomena and that fractal characteristics of the signal have *per se* physiological meaning.

Even if growing evidence has accumulated that circadian rhythm dysregulation not only is a risk factor for metabolic and cardiovascular diseases (Broussard and Van Cauter, 2016; Morris et al., 2016), but also contributes to neurodegenerative processes (Musiek et al., 2015), little attention has been paid to circadian rhythm modulations of brain dynamics in real clinical settings. Our data add evidence of circadian modulation in spectral and fractal features in healthy subjects. These results can help to characterize factors of intra-individual variability in describing brain dynamics and to personalize interventions or therapies in clinical applications. Indeed, if complexity of neuronal dynamics and long-term correlation of brain rhythms, factors related to the modality of neuronal responses to incoming input or sensory plasticity (Palva and Palva, 2011; Palva et al., 2013), changes during the daytime, it would be expected that the correct information on the time of the day when the individual state optimizes the individual response could be utilized to indicate the correct timing for a therapeutic or rehabilitative intervention. Therefore, the characterization of fractal phenomena can provide new psychophysical models (Zueva, 2015). In this direction, future studies are needed to underline alteration of circadian modulation of fractal features in neurological or psychiatric diseases, as well as to understand the link between fractal features, brain functions and behavior.

In conclusion, in our study differences in fractal features of rest EEG activity during the 4 daily times have been evidenced. Complexity and the persistence of temporal correlations of brain rhythms changes during daytime, parallel to changes in alertness and performance. The characterization of circadian modulations of fractal features may in future provide important information to build meaningful physiological models. Further studies under condition known to induce desynchrony amongst circadian oscillators are needed to disentangle the effects of circadian endogenous factors and homeostatic sleep pressure.

## Author contributions

PC, AQ, SC, and FZ designed the study, interpreted the data, and wrote the manuscript. PC and FZ carried out the experiments. PC, SC, and FZ analyzed the data. All of the authors participated in drafting the work and agreed on the final version of the manuscript.

### Conflict of interest statement

The authors declare that the research was conducted in the absence of any commercial or financial relationships that could be construed as a potential conflict of interest.

## Footnotes

^1^ActiGraph. Available online at: https://www.actigraphcorp.com/ (Accessed July 24, 2018).
